# Brain injury biomarkers do not predict delirium in acutely ill older patients: a prospective cohort study

**DOI:** 10.1038/s41598-023-32070-0

**Published:** 2023-03-27

**Authors:** Júlio César Garcia de Alencar, Flávia Barreto Garcez, Agnes Araujo Sardinha Pinto, Lucas Oliveira Junqueira e Silva, Lucas de Moraes Soler, Shirley Steffany Muñoz Fernandez, Victor Van Vaisberg, Luz Marina Gomez Gomez, Sandra Maria Lima Ribeiro, Thiago Junqueira Avelino-Silva, Heraldo Possolo Souza

**Affiliations:** 1 Disciplina de Emergências Clínicas, Faculdade de Medicina, Universidade de São Paulo, São Paulo, Brazil; 2 Hospital Universitário, Departamento de Medicina, Universidade Federal de Sergipe, São Cristóvão, Brazil; 3 Hospital de Cínicas de Porto Alegre, Universidade Federal do Rio Grande do Sul, Porto Alegre, Brazil; 4 Disciplina de Nefrologia, Faculdade de Medicina de Botucatu, Universidade Estadual de São Paulo, São Paulo, Brazil; 5 Departamento de Nutrição, Faculdade de Saúde Pública, Universidade de São Paulo, São Paulo, Brazil; 6 Faculdade Israelita de Ciências da Saúde Albert Einstein, Hospital Israelita Albert Einstein, São Paulo, Brazil; 7 Curso de Medicina, Faculdade de Odontologia de Bauru, Universidade de São Paulo, 9, Dr. Octávio Pinheiro Brisolla, Bauru, SP 17012-901 Brazil

**Keywords:** Neuroscience, Blood-brain barrier, Cell death in the nervous system, Neuronal physiology

## Abstract

Delirium is a common, serious, and often preventable neuropsychiatric emergency mostly characterized by a disturbance in attention and awareness. Systemic insult and inflammation causing blood–brain-barrier (BBB) damage and glial and neuronal activation leading to more inflammation and cell death is the most accepted theory behind delirium's pathophysiology. This study aims to evaluate the relationship between brain injury biomarkers on admission and delirium in acutely ill older patients. We performed a prospective cohort study which analyzed plasma S100B levels at admission in elderly patients. Our primary outcome was delirium diagnosis. Secondary outcomes were association between S100B, NSE and Tau protein and delirium diagnosis and patients’ outcomes (admissions to intensive care, length of hospital stay, and in-hospital mortality). We analyzed 194 patients, and 46 (24%) developed delirium, 25 on admission and 21 during hospital stay. Median of S100B at admission in patients who developed delirium was 0.16 and median was 0.16 in patients who didn’t develop delirium (*p*: 0.69). Levels S100B on admission did not predict delirium in acutely ill elderly patients.

Trial registration: The study was approved by the local institutional review board (CAPPESq, no. 77169716.2.0000.0068, October 11, 2017) and registered in Brazilian Clinical Trials Registry (ReBEC, no. RBR-233bct).

## Introduction

Delirium is a common, serious, and often preventable neuropsychiatric emergency that is characterized by a disturbance in attention and awareness^1,2^. It represents an acute and severe brain dysfunction, and it is associated with increased hospital and Intensive Care Unit (ICU) length of stay, persistent cognitive decline, and increased mortality^3^.

Systemic insult and inflammation causing blood–brain-barrier (BBB) damage, and glial and neuronal activation leading to more inflammation and cell death is the most accepted theory behind delirium's pathophysiology^4^. In addition to cognitive tests^5^, several plasma biomarkers and cytokines have been previously studied for delirium diagnosis^6^. Promising biomarkers are S100 calcium binding protein B (S100B) which is expressed by astrocytes and not only reflects cell death, but also BBB integrity and permeability; neuron-specific enolase (NSE) an isoenzyme highly specific to neurons, a biomarker of hypoxic brain damage and a marker of poor outcome after cardiac arrest; and Tau protein which maintains microtubules stability in axons and relates to forms of cognitive-impairment^7–9^. There are, however, many gaps in the literature to fully understand how these molecules interact and how they are associated with delirium occurrence^10,11^. Specifically, data on S100B are conflicting, since some studies have shown that patients with delirium had a higher serum level of S100B, and other studies have shown no association between this protein and delirium or other adverse outcomes^10^. Furthermore, there are no studies evaluating the association between inflammatory and brain-related biomarkers with Emergency Department (ED) delirium^11,12^.

Our primary goal was to evaluate S100B levels on admission and their association with delirium occurrence in acutely ill older adults. We also aimed to evaluate the association between S100B, NSE, Tau and cytokine panel (IL-1B, IL-4, IL-10, TNF-α and IFN-γ) with delirium. We hypothesized that increased levels of S100B, NSE and Tau would be associated with an increased risk of delirium.

## Methods

### Design, setting, and population

We prospectively screened patients admitted to the ED of a tertiary university hospital between September 30, 2019, and March 17, 2020. Hospital das Clínicas da Faculdade de Medicina da Universidade de São Paulo is a 2200-bed hospital located in Sao Paulo, Brazil, dedicated to the care of high-complexity medical and surgical patients. This report is published in accordance with the STROBE guideline and recommendations^13^.

Eligible patients were 65 years or older and hospitalized for less than 24 h. We excluded candidates according to the following criteria: (a) previous hospitalization in the 30 days preceding admission; (b) hospitalization for end-of-life care; and (c) expected hospital discharge in 48 h or less.

The study was approved by the local institutional review board (Comissão de Ética para Análise de Projetos de Pesquisa do HCFMUSP [CAPPESq], no. 77169716.2.0000.0068, October 11, 2017) and registered in Brazilian Clinical Trials Registry [(ReBEC), no. RBR-233bct)]. We obtained written informed consent from all participants or their legal representatives and used REDCap® (Research Electronic Data Capture) resources to secure and manage all study-related data^14^.

### Baseline characteristics

Trained investigators completed the study interviews and assessments using standardized REDCap forms. We collected baseline sociodemographic and clinical data including age, sex, literacy level, medical history, Charlson comorbidity index (Charlson)^15^, frailty status using the FRAIL scale^16^, polypharmacy (chronic use of five or more medications), and admission diagnoses. We performed functional and cognitive assessments using the activities of daily living (ADL) and the 10-point Cognitive Screener (10-CS) scales, respectively^17^.

### Delirium assessments

We completed the Confusion Assessment Method (CAM) algorithm^5^ twice daily to detect delirium. We performed the first assessment in the ED, and the following evaluations in Wards or ICUs, according to patients’ allocation. Our standardized interview protocol incorporated a brief neuropsychiatric anamnesis, cognitive screening (10-CS), attention testing (days of the week backwards and vigilance A test)^18^, level of consciousness assessment (Richmond Agitation and Sedation Scale [RASS])^19^, and electronic medical record revisions^20^. Delirium episodes were considered resolved if the patient was non-delirious for two consecutive evaluations.

Although our raters attended training sessions before the study initiation, which included simulations and bedside evaluations, and we achieved high interrater reliability levels for CAM-based delirium diagnosis (> 95%), whenever our raters were uncertain regarding the presence of delirium, two experienced physicians (JCGA and FBG) repeated or reviewed the assessments to confirm the final diagnosis.

### Blood samples

We collected the following laboratory tests upon study inclusion: blood count, C-reactive protein, platelets, creatinine, blood urea nitrogen, bilirubin, inflammatory biomarkers (IL-1B, IL-4, IL-10, INF-g and TNF-α and neuronal injury biomarkers (S100B, Neuron Specific Enolase and Tau protein).

Three registered nurses performed the sampling while patients were in the ED, which consisted of 30 ml of blood collected by venipuncture. Blood samples used for brain injury biomarkers analysis were immediately centrifuged for 10 min, and plasma was preserved at − 20 °C for up to 48 h before being transferred to a − 80 °C freezer for long-term storage and further processing.

We measured cytokine plasma levels using the magnetic bead immunoassay Milliplex® and the MAGPIX® System (Merck Millipore, USA).

**Figure 1:**
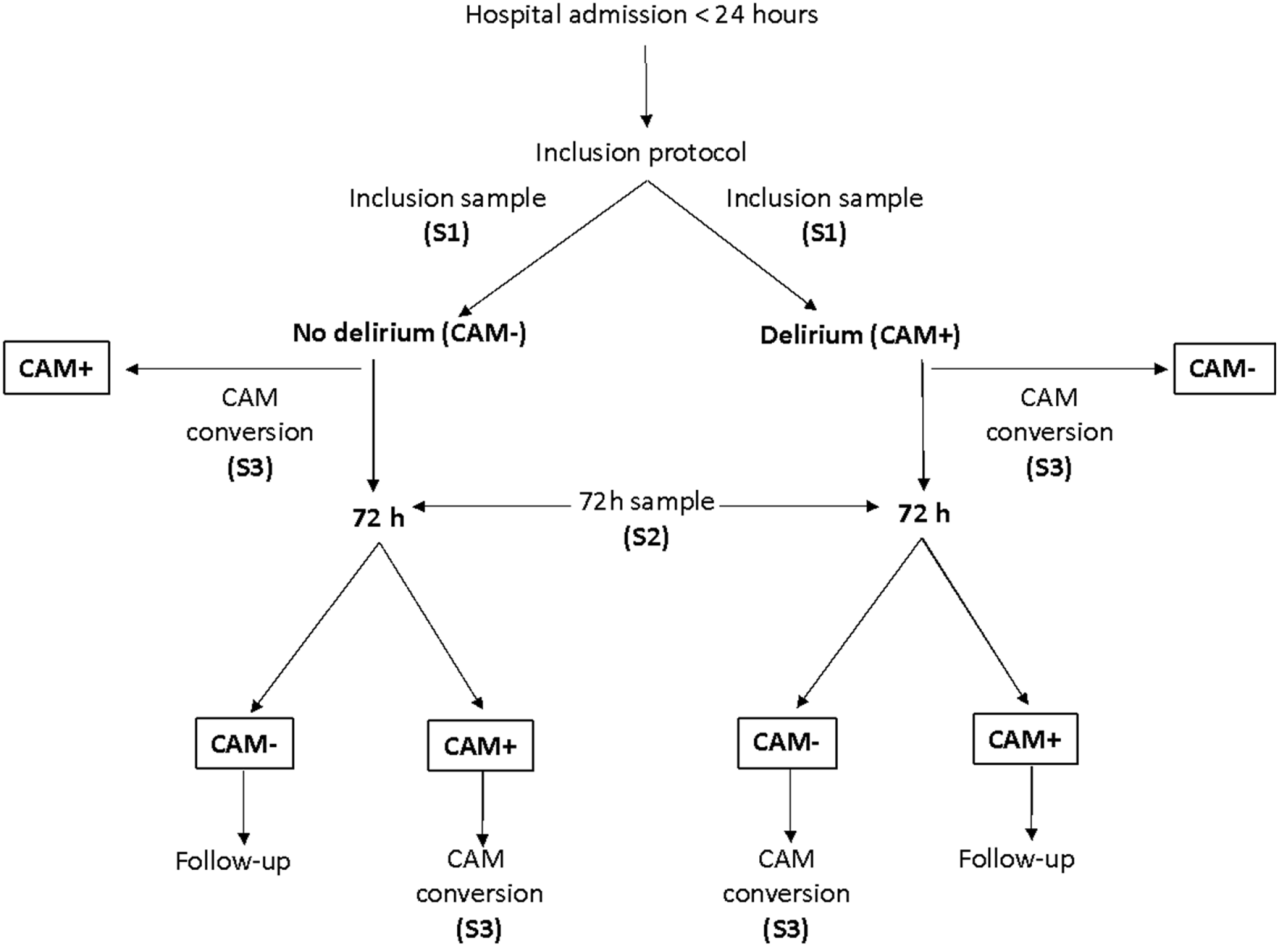
Flowchart of study procedures.

The sampling procedures were performed on inclusion (S1) and repeated 72 h after inclusion (S2)^21^. Participants who were discharged or died within 72 h of admission, or refused to provide additional samples, were not punctured again. We obtained a third sample (S3) from participants who converted either from a negative to positive CAM (incident delirium) or from a positive to negative CAM (delirium resolution) after S2 (Fig. 1).

### Statistical analyses

Our primary outcome was the overall occurrence of delirium based on the CAM criteria. Secondary outcomes were association between S100B, NSE and Tau protein and delirium diagnosis and patients’ outcomes (admissions to intensive care, length of hospital stay, and in-hospital mortality).

We used a convenience sample, which limited the total number of enrolled patients^22^. Despite our ED providing medical care to 800 elderly patients monthly and 30% of them being eligible for hospitalization, most of these patients are transferred from other less complex hospitals and had been hospitalized for more than 24 h at the time of recruitment. Therefore, 200 patients were predicted to be enrolled for 6 months, with the expectation of 7–10 eligible patients per week. We finished our recruitment a month ahead of schedule because of the beginning of the COVID-19 pandemic.

All enrolled patients were included in the analysis of primary and secondary outcomes on an intention-to-treat basis. We initiated the analysis using the Shapiro–Wilk test to determine which variables were normally and non-normally distributed, especially results of S100B. Then, we performed analysis according to delirium occurrence using unpaired *t-tests* or Kruskal–Wallis for normal and non-normal variables respectively, and categorical variables were analyzed using Pearson’s χ^2^ test.

All analyses were performed with Stata software, version 10.

### Ethics approval and consent to participate

This study was approved by the São Paulo University’s Research Ethics Committee *Comissão de Ética para Análise de Projetos de Pesquisa do HCFMUSP* (CAPPESq), no. 77169716.2.0000.0068 on October 11, 2017, and registered in Brazilian Clinical Trials Registry (ReBEC, no. RBR-233bct). All participants or legal representatives provided written informed consent prior to enrolment in the study.

## Results

### Patient characteristics

**Figure 2:**
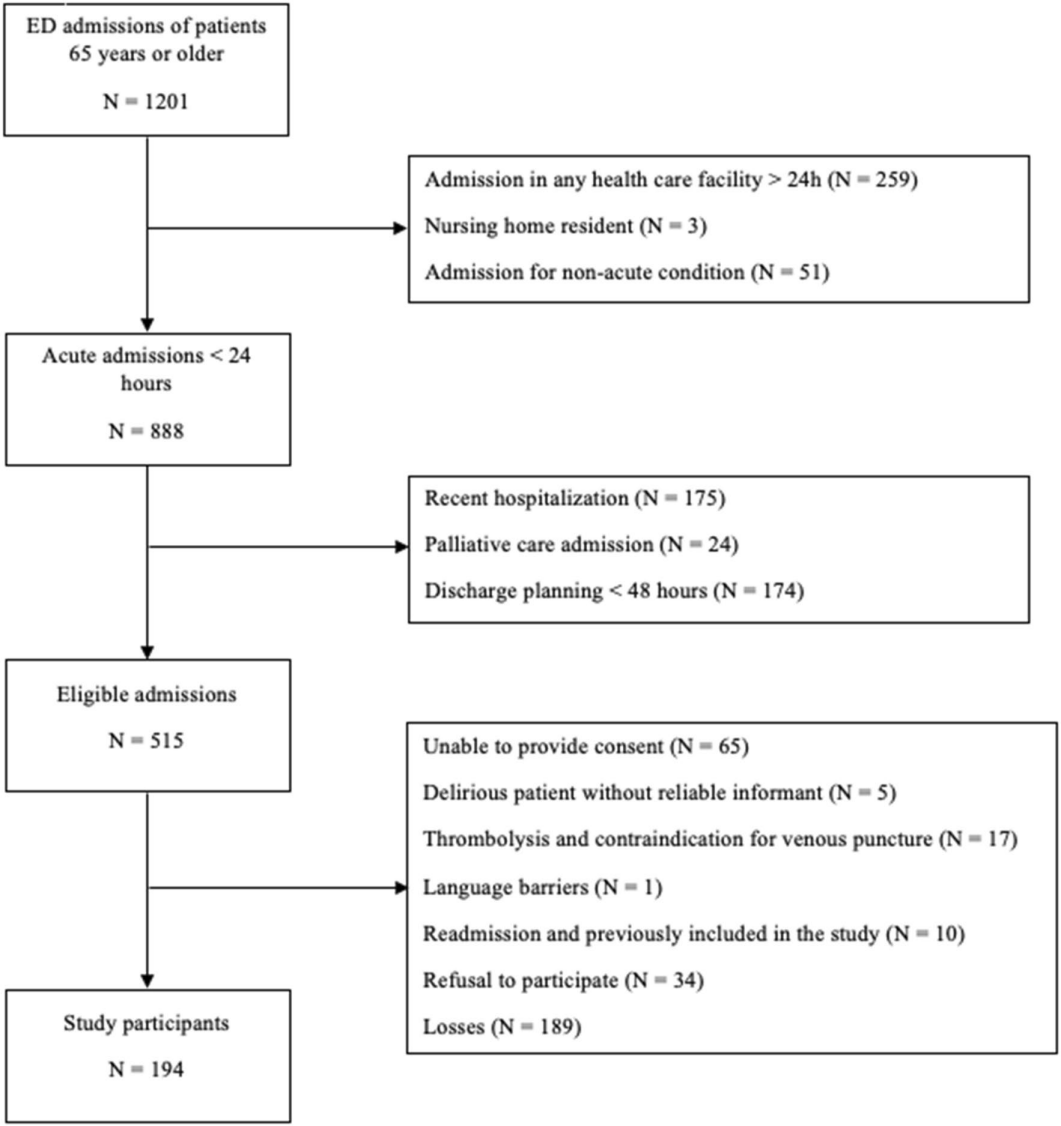
Flowchart illustrating the enrollment of the study population.

We included 194 participants (Fig. 2 and Table 1). They were mostly female (60.52%), had a mean age of 74.7 (± 7.4) years, and were hospitalized for a median 8 (4–15) days. The main cause of admission was sepsis (49 patients, 25%), followed by cardiovascular disease (20%), cerebrovascular disease (14%) and abdominal surgical conditions (10%). Overall, 72% were referral to ward, and 28% of our sample required ICU admission, with a stay of 4 (2–7) days, 22% required invasive mechanical ventilation and 14% died. (Supplementary Appendix: Outcomes). We detected delirium in 46 individuals (24%)—25 on admission (prevalent delirium) and 21 during hospital stay (incident delirium). Delirium patients were significantly older and had more history of cerebrovascular accident or dementia. Furthermore, delirium patients had longer days of hospitalization (mean 10 vs 7, *p* = 0.0212), increased need of ICU care (41.3% vs 24.3%, *p* = 0.0256), and increased mortality (32.61% vs 7.43%, *p* < 0.0001) (Table 1).

**Table 1:** Patient characteristics.

Characteristics and comorbidities	Total (N = 194)	Delirium (N = 46)	Non delirium (N = 148)	*p* value
Age, y (IQ)	73.5 (69–79)	77.5 (73–85)	72 (68–78)	< 0.0001*
Male sex, N (%)	39.1	52.1	35.1%	0.03**
Years of schooling	4 (2–8)	4 (3–8)	5 (2–8)	0.63
Previous diseases				
Systemic arterial hypertension	70.6	73.9	69.5	0.57
Diabetes	35.1	43.5	32.4	0.17
Obesity	1.0	0	1.3	0.43
Dyslipidemia	21.1	28.3	18.9	0.17
Chronic kidney disease	11.3	6.5	12.8	0.24
Liver failure	5.1	2.2	6.1	0.29
Stroke	14.9	30.4	10.1	0.0007*
Transient ischemic event	1.5	2.2	1.3	0.69
Dementia	2.1	6.5	0.7	0.01**
Depression	6.2	10.9	4.7	0.13
Parkinson’s disease	0.51	2.2	0	0.07
Acute myocardial infarction	15.5	8.7	17.6	0.14
Cardiac insufficiency	9.2	4.3	10.8	0.18
Active cancer	5.7	4.3	6.1	0.65
Clinical outcomes				
Days of hospitalization	8 (4–15)	10 (5–25)	7 (4–14)	0.02*
ICU care	28.3	41.3	24.3	0.02*
Mortality	13.4	32.6	7.4	< 0.0001*

Variables are expressed in percentage (%), except for age, years of schooling and days of hospitalization which are expressed in median (interquartile range).

*p values were calculated using the non-parametric Mann–Whitney test for continuous quantitative variables. **p values were calculated using Chi-squared test for categorical variables.

### Association of S100B and other biomarkers with delirium occurrence

Plasma S100B and other biomarkers concentrations at ED admission were not associated with an increased risk of delirium diagnosis during hospitalization (Table 2) (Supplementary Appendix: Results Biomarkers).

**Table 2:** Results of biomarkers at admission in delirium and non-delirium patients.

	Total (N = 194)	Delirium (N = 46)	Non delirium (N = 148)	*p* value
S100B	0.16 (0.13–0.21)	0.16 (0.12–0.22)	0.16 (0.13–0.21)	0.69
NSE	1.70 (1.10–2.65)	1.81 (1.10–2.70)	1.69 (1.10–2.61)	0.57
Tau	61.04 (41.49–96.92)	68.80 (42.43–96.92)	58.93 (41.32–97.57)	0.61
IL 1 beta	0.86 (0.77–0.96)	0.86 (0.77–0.96)	0.86 (0.77–0.96)	0.49
IL 4	3.34 (1.95–3.39)	3.39 (1.95–3.39)	2.67 (1.95–3.39)	0.52
IL 10	3.92 (2.15–11.24)	3.39 (2.07–10.30)	4.61 (2.40–12.33)	0.41
IFN gamma	1.78 (1.47–2.45)	1.78 (1.47–2.63)	1.78 (1.47–2.10)	0.15
TNF alpha	22.19 (12.41–34.02)	22.75 (12.24–34.02)	20.84 (13.77–34.62)	0.85

In a post-ROC analysis, only patients at risk of developing delirium were evaluated (i.e., those without delirium at enrollment), and 25 patients admitted with CAM positive were excluded. Table 3 compares plasma levels of S100B, NSE and Tau between patients who developed and those who did not develop delirium during hospitalization.

**Table 3:** Results of biomarkers at admission in patients before delirium and non-delirium patients.

	Before Delirium (N = 21)	No Delirium (N = 148)	*p* value
S100B	0.15 (0.10–0.20)	0.16 (0.13–0.21)	0.44
NSE	1.67 (0.98–2.51)	1.69 (1.10–2.61)	0.87
Tau	65.84 (36.81–94.23)	58.93 (41.32–97.57)	0.73

### Association of S100B and other biomarkers with delirium diagnosis

Plasma S100B, NSE and Tau were measured before and during delirium in 21 patients with delirium during hospital stay (delirium incidence). Levels did not differ significantly in the two groups (Table 4).

**Table 4:** S100B, NSE and Tau before and during delirium.

	Before Delirium (N = 21)	Delirium (N = 21)	*p* value
S100B	0.15 (0.10–0.20)	0.15 (0.11–0.21)	0.67
NSE	1.67 (0.98–2.51)	1.73 (1.24–3.47)	0.88
Tau	65.84 (36.81–94.23)	64.83 (47.18–127.77)	0.13

### Association of S100B and other biomarkers with outcomes

Plasma S100B, NSE and Tau protein levels were not significantly associated with outcomes (Table 5).

**Table 5:** S100B, NSE and Tau and outcomes.

	Ward (N = 139)	ICU admission (N = 55)	p value	Discharge (N = 166)	Death (N = 28)	*p* value
S100B	0.16 (0.13–0.22)	0.16 (0.13–0.21)	0.92	0.16 (0.13–0.21)	0.16 (0.12–0.27)	0.94
NSE	1.67 (1.08–2.62	1.79 (1.11–2.80)	0.64	1.71 (1.11–2.65)	1.64 (1.10–2.65)	0.92
Tau	59.27 (40.00–96.88)	65.84 (43.04–99.28)	0.66	61.04 (41.14–98.20)	63.70 (43.31–93.60)	0.58

## Discussion

Our study demonstrated that serum S100B on admission were not associated with delirium in acutely ill older patients.

Previous studies have demonstrated an association between plasma levels of S100B and delirium occurrence^23,24^ and duration^25^ in ICU patients. The mechanism behind the increase of S100B in blood is uncertain. Authors have hypothesized that cerebral or extra-cerebral cellular damage caused by multiple different mechanisms (such as hypoxia) could lead to neuroinflammation and subsequent increased permeability of the BBB, ultimately upregulating S100B production by astrocytes^26^. These results are not unanimous, and previous studies with ICU patients did not confirm the association between plasma levels of S100B and the occurrence of delirium^27^.

Our results are consistent with McNeil et al. in Delineate study, which did not demonstrate an association between serum S100B levels and delirium duration^12^. We consider that delirium’s pathogenesis is multifactorial, probably include systemic inflammation and endothelial dysfunction, but this association may be modified by baseline patient’s conditions.

On the other hand, van Munster et al*.* demonstrated that plasma levels of S100B were higher in critically ill elderly patients during and after delirium than in patients without delirium^28^. These results were not reproduced in our patients. The authors reported that S100B levels remain high after delirium, which could indicate an active stimulation of astrocytes or an increase of BBB’s permeability. It is worth mentioning that they did not assess the role of S100B in delirium occurrence and measurements S100B occurred 48 h after admission.

Interestingly, van Munster also studied the role of S100B in predicting delirium, and demonstrated that among patients undergoing surgery, S100B levels were higher in those who developed delirium than in patients who did not^29^. These findings were not reproduced by the same author when she evaluated the preoperative role of S100B in cerebrospinal fluid (CSF)^30^.

Our study does not demonstrate a statistically significant association between plasma NSE or Tau levels at admission in ED and occurrence of delirium during hospitalization. As far as we are aware, this is the first study to analyze these proteins while patients were in the ED. Our findings differ from previous studies that demonstrated this association in clinical and surgical patients in the ICU setting^24,27,31,32^.

### Limitations

Several limitations need to be acknowledged. First, S100B values were measured in peripheral blood and may not necessarily correspond to values in the brain. Under normal conditions, serum S100B content is lower than that in CSF^33^. However, we were searching a feasible and reproducible serum biomarker. Second, there were a sizeable number of patients who were not enrolled during the study period. Our research team recruited patients daily in the morning, however, some patients stayed a few hours in the ED before being transferred to wards or ICUs. Our sample size could associate our results with a type 2 error. Nevertheless, with a sample size of almost 200 patients, we believe that the effect size would be too small and even irrelevant to had not been established in this pragmatic ED study. Finally, probably not all delirium results from neuronal injury or BBB damage. There are other causes of delirium, including sepsis-associated encephalopathy and drug withdrawal. This means that S100B may not raise in all delirium patients and further research should explore the association between S100B, NSE, Tau and subsequent cognitive decline.

## Conclusions

S100B and other brain injury biomarkers measured on admission are not associated with delirium in acutely ill older patients. Future studies with others and serial biomarker measurements throughout delirium’s course and long-term cognitive outcome are needed to better clarify these relationships.

## Supplementary Information

Supplementary Information 1.

Supplementary Information 2.

## Data Availability

All data generated or analyzed during this study are included in this published article (Supplementary files “Results Biomarkers” and “Outcomes”).

## Abbreviations

10-CS: 10-Point Cognitive Screener
ADL: Activities of daily living
BBB: Blood–brain-barrier
CAM: Confusion assessment method
CSF: Cerebrospinal fluid
ED: Emergency department
ICU: Intensive care unit
NSE: Neuron-specific enolase
RASS: Richmond Agitation and Sedation Scale
REDCap: Research Electronic Data Capture
S100B: S100 calcium binding protein B
